# Difficulties in Keeping an Intimate Relationship and Singlehood

**DOI:** 10.1177/14747049251377388

**Published:** 2025-09-08

**Authors:** Menelaos Apostolou, Timo Juhani Lajunen

**Affiliations:** 1 Department of Social Sciences, 121343 University of Nicosia, Nicosia, Cyprus; 2 Department of Psychology, 8018 Norwegean University of Science and Technology, Trondheim, Norway

**Keywords:** singlehood, difficulties in keeping an intimate relationship, between-relationships singlehood, voluntary singlehood, involuntary singlehood

## Abstract

An increasing number of people are single, meaning that they do not have an intimate partner. Existing research has focused on identifying the difficulties that people face in attracting mates. In the present paper, we propose that another factor contributing to singlehood is experiencing difficulties in maintaining intimate relationships. By analyzing data collected from 1099 Greek-speaking participants, we found that individuals who experienced greater difficulties maintaining intimate relationships were more likely to be either between-relationships single or voluntarily single rather than in an intimate relationship. For women specifically, higher scores in this dimension were also associated with a greater probability of being in an intimate relationship than being involuntarily single. Additionally, we found that the association between difficulties in maintaining an intimate relationship and relationship status was linear for men—the relationship between the two variables can be pictured as straight line—but curvilinear—the relationship can be pictured as an inverted U-shaped curve—for women.

Not infrequently, people experience difficulties attracting intimate partners and consequently remain single, at least for some period of time (Apostolou, 2015). The rising rate of singlehood (Adamczyk & Trepanowski, 2023) has prompted research aimed at identifying and understanding the contributing factors (e.g., Apostolou & Michaelidou, 2023). However, difficulties in attracting partners are not the sole reason behind singlehood; individuals may also encounter challenges maintaining intimate relationships. The purpose of the current study is to examine the association between the degree of difficulty individuals experience in maintaining intimate relationships and their relationship status. We begin our analysis by addressing the ultimate or evolutionary reasons underlying the phenomenon of singlehood (for different theoretical perspectives on understanding singlehood see DePaulo, 2007; Girme et al., 2023; Kislev, 2019).

## The Mismatch Problem and Singlehood

The adaptations organisms possess interact with specific aspects of their environment in ways that increase the likelihood that the genes coding for these adaptations are represented in future generations—a concept known as fitness (Irons, 1998). When environmental conditions change, these adaptations could become less effective at performing their original function, triggering selective pressures aimed at restoring their effectiveness by modifying these mechanisms to deal with the new conditions. However, such evolutionary modifications require time to complete, leaving individuals temporarily relying on mechanisms that are suboptimal for addressing new environmental challenges. This phenomenon is known as the mismatch problem (Li et al., 2018), which is likely to constitute a key reason why individuals encounter difficulty attracting mates (Apostolou, 2015).

More specifically, anthropological, physiological, historical, and phylogenetic evidence indicates that in ancestral pre-industrial human societies, long-term mating was predominantly arranged by parents (Apostolou, 2014; Walker et al., 2011) or was forced by dominant males (Puts, 2016), with people having also some freedom to exercise independent mate choice (Apostolou, 2017). This pattern contrasts sharply with contemporary post-industrial societies, where mate choice is exercised freely. This mismatch between ancestral and modern contexts likely influenced various mechanisms involved in mate attraction, such as flirting skills. As a result, individuals may face difficulty when attracting mates independently, leading them to remain single (Apostolou, 2015). Nevertheless, there are also reasons to believe that the mismatch problem has negatively impacted mechanisms associated with maintaining intimate relationships, contributing further to singlehood.

In greater detail, it has been argued that the ancestral conditions for maintaining intimate relationships differed substantially from contemporary circumstances (Apostolou & Wang, 2020). Firstly, in pre-industrial societies, social protection systems do not exist, causing individuals to depend heavily upon their partners for support and protection, thereby creating strong incentives to remain in a relationship. Conversely, in contemporary post-industrial societies, people are often financially independent and possess alternative social and institutional protections in times of hardship. This mismatch is likely to have influenced relationship-related mechanisms, including relationship enthusiasm and the mating effort required to maintain the relationship. Specifically, the strong incentives in ancestral contexts to remain partnered—due to needs for protection and support—reduced the necessity for high levels of relationship enthusiasm, and mating effort to keep an intimate relationship. Today, these incentives have considerably diminished, and consequently, individuals often experience reductions in relationship enthusiasm, and the commitment to sustained mating effort, complicating the maintenance of long-term intimate relationships (Apostolou & Wang, 2020).

Furthermore, in ancestral pre-industrial societies, where human rights protection was weak or nonexistent, strategies involving physical coercion to influence partners into complying—including staying in the relationship—were probably effective, particularly for men. In more specific terms, men are, on average, physically larger and stronger than women (Puts, 2016), allowing them historically to leverage this physical advantage. For example, men could threaten physical punishment if their partners interacted with other men or attempted to leave the relationship. Such coercive strategies could have proven effective under ancestral conditions, and selection pressures may thus have predisposed men toward employing them (Buss, 2021). Conversely, this predisposition has negative implications on people's capacity to keep an intimate relationship in contemporary post-industrial societies, where human rights are strongly protected and physical aggression is no longer tolerated.

## Difficulties in Keeping Intimate Partners and Singlehood

Our hypothesis is that people who experience difficulty maintaining intimate relationships will have an increased probability of being single. Yet, singlehood is not a homogeneous category (Girme et al., 2023). Three main categories of single individuals have been identified: Involuntarily singles—those who desire an intimate relationship but face difficulties attracting mates; voluntarily singles—those who prefer not to be in an intimate relationship; and between-relationships singles—those whose previous relationship recently ended and who are in the process of attracting a new partner (Apostolou & Wang, 2019). We predict that difficulties maintaining intimate partners will be associated specifically with voluntary and between-relationships singlehood.

In particular, individuals who struggle to maintain intimate relationships are likely to experience relationship failure frequently, thus increasing their likelihood of being between-relationships singles compared to those who do not face such difficulties. In other words, they would frequently find themselves in situations in which one relationship has ended, and they are subsequently seeking a different intimate partner. Moreover, the termination of an intimate relationship typically provokes intense negative emotions, such as sadness, anger, and despair (Apostolou et al., 2025). Consequently, individuals who repeatedly experience difficulties maintaining relationships will have an elevated risk of relationship failure and thus recurring negative emotional experiences. These emotional burdens may discourage individuals from actively seeking new relationships, instead motivating them to remain voluntarily single to avoid repeating these experiences. Furthermore, involuntary singlehood predominantly arises from the difficulties individuals face in attracting mates. As such, difficulties in maintaining relationships may not necessarily predict this type of singlehood. Nevertheless, difficulties in attracting mates and maintaining relationships may overlap to some extent. If this overlap exists, the latter difficulties could also be associated with involuntary singlehood. Since the degree of overlap between these two types of difficulties is unknown, we will not hypothesize a specific direction for the association between difficulties in maintaining intimate relationships and involuntary singlehood.

## Methods

### Participants

For the purposes of the current study, we conducted further analysis on the dataset regarding difficulties in maintaining intimate relationships collected by Apostolou and Wang (2020). Data were collected as follows: Four research assistants recruited adult volunteers (18 years or older) for a study on romantic relationships, conducted in Greece and the Republic of Cyprus over four months. Participants provided consent before completing a survey, which they sealed in an unmarked envelope upon completion. No payment was offered for participation. The dataset consisted of a sample of 1099 participants who were asked to rate how much they felt restricted by 78 difficulties in maintaining an intimate relationship. Answers were recorded using a five-point Likert scale (1 = strongly disagree, 5 = strongly agree). Sample items include “I do not take into consideration my partner's needs,” “Bad sex,” “I often become violent to my partner,” and “My partner does not have good relationships with my parents and relatives.” Using exploratory factor analysis the original study classified the 78 items in 12 broader difficulties in maintaining intimate relationships.

Participants’ relationship status was also recorded (single, in a relationship, married). Those who identified as single were subsequently asked to clarify their answers by choosing from the following options: (a) Involuntarily single: “I want to be in a relationship but I face difficulties attracting intimate partners”; (b) Voluntarily single: “At the moment, I do not have an interest in being in an intimate relationship”; (c) Between-relationships single: “My intimate relationship has recently ended, and I have not yet found another intimate partner.” The mean age of female participants was 32.7 years (*SD* = 11.9), while the mean age of male participants was 33.5 years (*SD* = 10.6). In terms of relationship status, 38.6% of participants were married, 30.1% were in a relationship, 14.4% were voluntarily single, 11.7% were between-relationships single, and 5.2% were involuntarily single. Additionally, 39.9% of participants indicated having children. All data are available here: https://osf.io/q72dx/?view_only=74d50249e6d74fa4b635f74bc9c7a23a

### Data Analysis

Based on participants’ responses to the relationship status questions, we created a new variable “relationship status” with the following categories: involuntarily single, voluntarily single, between-relationships single, and in an intimate relationship. The “in an intimate relationship” category was formed by merging the original categories “in a relationship” and “married” and served as the reference category in the statistical analysis. Additionally, we created a new variable by averaging participants’ scores across all 78 items. This “difficulties” variable reflects the overall level of difficulties participants experience in maintaining intimate relationships. Preliminary analyses indicated that the association between relationship status and difficulties was linear among male participants and nonlinear (quadratic, of the form *x* + *x*^2^) among female participants. Accordingly, multinomial logistic regression analyses were conducted separately by sex. For male participants, the “difficulties” variable was entered as the primary independent variable. For female participants, both “difficulties” and “difficulties squared” were entered as independent variables. Participants’ age and parenthood status (having or not having children) were included as covariates in these analyses.

## Results

From Table 1, we observe that among female participants there was a curvilinear relationship between perceived difficulties in maintaining an intimate relationship and the likelihood of being between-relationships single as opposed to being in an intimate relationship. Specifically, starting from a low level of difficulty, increases in perceived difficulties were associated with a sharp rise in the probability of being between-relationships single rather than in an intimate relationship (positive linear term, *OR* = 62.3). However, this increase became progressively weaker at higher difficulty levels (negative quadratic term, *OR* = 0.52), resulting in a nonlinear, inverted U-shaped pattern. The estimated probability of being between-relationships single compared to being in an intimate relationship reached a maximum at a difficulty level of approximately 3.16 on the five-point scale. At this level—and given the intercept of −6.26—the probability of being between-relationships single (vs. being in an intimate relationship) was approximately 56.7%.

**Table 1.** The Effect of Difficulties in Keeping an Intimate Relationship on Relationship Status.

			Between-relationships single		Voluntarily single		Involuntarily single	
	Chi-square	*p*-Value	OR	*p*-Value	OR	*p*-Value	OR	*p*-Value
Women								
Difficulties	23.88	<.001	62.3	.005	305.0	<.001	40.8	.048
Difficulties squared	19.81	<.001	0.52	.014	0.37	.002	0.52	.070
Men								
Difficulties	14.85	.002	2.36	<.001	1.81	.013	1.09	.827

Note: Reference category is “In an intimate relationship.”

For men, we report the results of the analysis where the difficulties variable entered in both linear and quadratic forms.

To summarize this result, female participants reporting low perceived difficulty levels (scores around 1 to 2) had a relatively low likelihood of being between-relationships single. As perceived, difficulties increase toward moderate levels (near 3), the probability of being between relationships increases sharply. Beyond this threshold, further incremental increases in difficulty had limited or slightly diminishing effects on this probability. A similar curvilinear relationship was observed for voluntary singlehood among female participants. The probability of being voluntarily single, relative to being in an intimate relationship, peaked at a difficulty level of approximately 2.88, with a corresponding probability estimate of 29.6% at this level (intercept = −9.09). Likewise, for involuntary singlehood, the probability peaked at approximately 2.83 on the difficulty scale, with an estimated probability of 18.7% of being involuntarily single versus in an intimate relationship at this point (intercept = −6.73).

**Figure 1.**
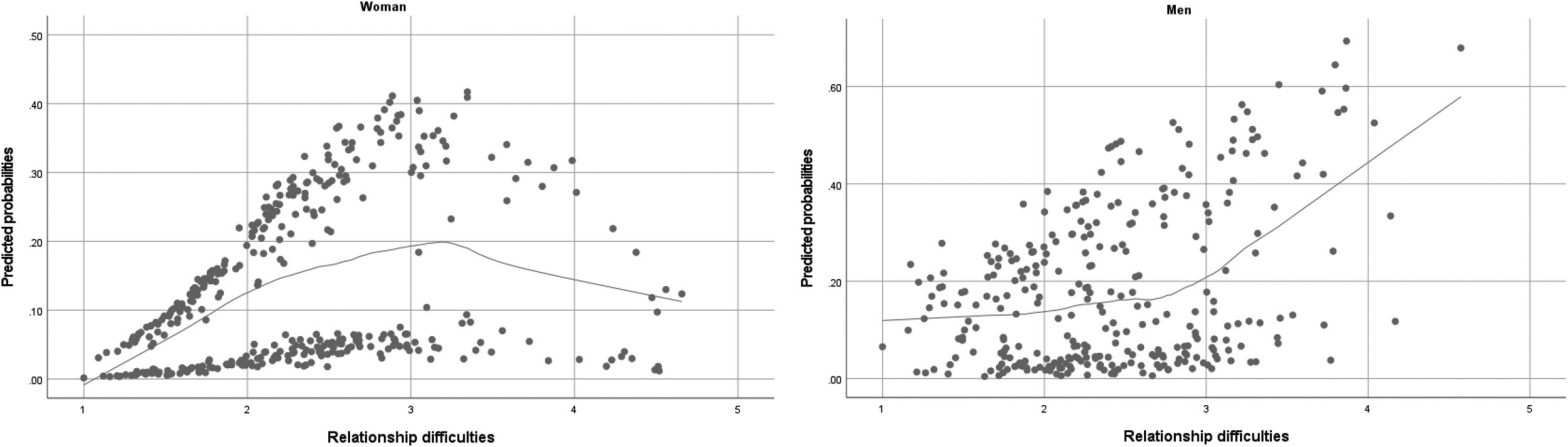
The Figure Above Presents Predicted Probability Curves for Women and Men for Between-Relationship Singlehood. For Women, as Difficulties Increase, the Probability of Being Single (vs. in an Intimate Relationship) Rises to a Point and then Declines. For Men, the Probability of Being Single Increases with Difficulties. Comparable Patterns were Observed for Voluntary Singlehood.

From Table 1, we also observe that among male participants each one-unit increase in perceived difficulties corresponds to a 2.36-fold increase in the probability of being between-relationships single rather than being in an intimate relationship. Additionally, among men, each one-unit increase in perceived difficulties corresponds to a 1.81-fold increase in the likelihood of being voluntarily single relative to being in an intimate relationship. On the other hand, perceived difficulties had no statistically significant effect on involuntary singlehood in male participants (see Figure 1 for a visual depiction of the observed associations).

## Discussion

In the present study, we examined the association between the difficulties individuals experience in maintaining intimate relationships and their relationship status. Consistent with our predictions, we found that participants who face greater difficulties in maintaining intimate relationships were more likely to be either between-relationships single or voluntarily single, rather than in an intimate relationship. Among women, higher scores in this dimension were also associated with an increased probability of being in an intimate relationship rather than involuntarily single. Additionally, we found that the association between relationship status and difficulty maintaining intimate relationships was linear for men and curvilinear for women.

Considering the latter finding in more detail, as men's difficulties in maintaining an intimate relationship increased, their probability of being single rose steadily, with the impact remaining relatively consistent across the scale of difficulties. On the other hand, for women, the effect was pronounced only up to around the mid-point of the difficulty scale, beyond which additional increases in difficulty had limited influence on their probability of being single. In practical terms, this finding indicates that a man scoring “4” on the difficulty scale will be considerably more likely to be single than a man scoring “3.” In contrast, a woman scoring “4” will differ only slightly in singlehood probability from a woman scoring “3.” Currently, we do not have an explanatory hypothesis to account for this observed sex difference, and this finding warrants further investigation.

With respect to involuntary singlehood, the effect for men was not statistically significant, while for women it was close to the significance level. This finding suggests that women who face difficulties in maintaining intimate relationships may also have an increased likelihood of involuntary singlehood. One possible explanation is that for women, difficulties in attracting intimate partners may overlap with difficulties in maintaining relationships; thus, difficulties in attraction could act as a proxy for difficulties in relationship maintenance. Stated differently, women in our sample who struggled to maintain relationships may concurrently encounter difficulties attracting intimate partners, which could predict involuntary singlehood. Future research should examine more closely the extent to which difficulties in attraction and relationship maintenance are linked, and whether this association differs between men and women.

A limitation of our study is that the current dataset relies on self-report instruments, which can be subject to biases, such as participants providing inaccurate or biased responses regarding their personal difficulties in maintaining intimate relationships. Furthermore, the cross-sectional study design prevents us from establishing causal relationships among the variables examined. Another limitation pertains to the cultural context, given that the study was confined to the Greek cultural setting. Thus, cross-cultural replication is essential to determine the extent to which the results generalize to different cultural contexts. Additionally, our study used an evolutionary perspective; other theoretical perspectives may also inform our understanding of the association between difficulties in maintaining intimate relationships and singlehood.

Research to date has predominantly concentrated on understanding the factors restricting people's ability to attract intimate partners, thereby explaining involuntary singlehood (Apostolou & Michaelidou, 2023). In our current study, we extended the argument by proposing that the mismatch problem similarly affects mechanisms involved in maintaining relationships, causing individuals to encounter difficulties in this area, consequently leading to singlehood. Our findings align with this hypothesis, demonstrating that increased difficulties in maintaining intimate relationships are indeed associated with singlehood, especially the between-relationships type. Future studies should further explore the association between difficulties in attracting mates, difficulties maintaining relationships, and their individual and combined roles in predicting singlehood. To conclude, people who face difficulties in maintaining intimate relationships are considerably more likely to be single, and future work needs to identify the factors that predict these difficulties and, consequently, singlehood.
